# Transfer of faeces in ulcerative colitis 2: improving efficacy – study protocol for a multicentre randomised controlled trial (TURN2 study)

**DOI:** 10.1136/bmjopen-2025-107097

**Published:** 2026-05-05

**Authors:** Mèlanie V Bénard, Mirjam JW Van Der Spek, Mark Davids, Caroline E Visser, Erwin G Zoetendal, Bente Rethans, Florine H Zwezerijnen-Jiwa, Marijn C Visschedijk, Bas Oldenburg, Rinse K Weersma, Cyriel Y Ponsioen

**Affiliations:** 1 Department of Gastroenterology and Hepatology, Amsterdam University Medical Centres, Amsterdam, The Netherlands; 2 Microbiota Center Amsterdam (MiCA), Amsterdam UMC Locatie AMC, Amsterdam, The Netherlands; 3 Department of Experimental Vascular Medicine, Amsterdam UMC Location AMC, Amsterdam, The Netherlands; 4 Department of Medical Microbiology and Infection Prevention, Amsterdam UMC, Amsterdam, The Netherlands; 5 Laboratory of Microbiology, Wageningen University & Research, Wageningen, The Netherlands; 6 Amsterdam Gastroenterology Endocrinology and Metabolism, Tytgat Institute for Liver and Intestinal Research, Amsterdam, The Netherlands; 7 Gastroenterology and Hepatology, University Medical Centre Groningen, Groningen, The Netherlands; 8 Department of Gastroenterology and Hepatology, University Medical Centre Utrecht, Utrecht, The Netherlands

**Keywords:** Clinical Trial, Inflammatory bowel disease, Randomized Controlled Trial

## Abstract

**Introduction:**

The interaction between the gut microbiota and the host immune system is implicated in the pathogenesis of inflammatory bowel disease, including ulcerative colitis (UC). Targeting the gut microbiota with faecal microbiota transplantation (FMT) from a healthy donor has shown promise in inducing remission in patients with active UC. However, mixed results and protocol heterogeneity have limited its practical application. Our previous Transfer of Faeces in Ulcerative Colitis; Restoring Homeostasis (TURN) trial found a correlation of clinical response with specific strains and butyrate production. Since most gut microbes, including many butyrate producers, are anaerobes, anoxic processing of donor stool may be essential to increase efficacy of FMT in UC. This trial aims to enhance FMT efficacy by applying strict anoxic processing, selecting donors based on microbial composition and using repetitive dual-route administration.

**Methods and analysis:**

This randomised, double-blind, placebo-controlled, multicentre study evaluates the efficacy of strictly anoxic prepared donor FMT compared with anoxic prepared autologous FMT in patients with mild to moderate active UC. An open-label extension option is available for non-responders in the autologous arm. Included patients will receive 4 weekly FMTs, comprising two double-route administrations (nasoduodenal administration combined with enema) and two single enemas. Donors are selected based on their microbiota profile, informed by our previous TURN trial and literature. A total of 76 patients evaluable for the primary endpoint will be included. The primary endpoint is steroid-free clinical and endoscopic remission at week 8, assessed by the adapted Mayo score. An interim analysis will be conducted midway through the study by a Data Safety Monitoring Board to monitor efficacy and safety. Other outcomes of this study include the evaluation of clinical, endoscopic and histological response. In addition to clinical results, this study aims to provide valuable insights into specific microbial strains, metabolites and mechanisms correlated with response, aiding in the development of future microbial therapies.

**Ethics and dissemination:**

Ethics approval was obtained from the medical ethics committee of the Amsterdam University Medical Centre in the Netherlands (reference number 2018_057). All participants will provide written informed consent. The results of the trial will be disseminated through publication in a peer-reviewed journal and presentations at (inter)national conferences.

**Trial registration number:**

Prospectively registered in May 2018 in the Dutch Trial Register (NTR/LTR) as NL7770. Assigned NL-OMON52507 following the transition of the Dutch Trial Register to the Overview of Medical Research in the Netherlands. Also registered at ClinicalTrials.gov (NCT05998213).

**STRENGTHS AND LIMITATIONS OF THIS STUDY**

- This is one of the first multi-centre trials applying anoxic processed faecal microbiota transplantation (FMT) from microbiota-selected faeces donors, with a long follow-up period of 1 year.
- In addition to evaluating the efficacy of this adjusted FMT protocol, the study will provide insights into FMT’s mechanism of action via microbiome and metabolome analyses.
- A blinded critical event committee is established to enhance reliability of the primary endpoint.
- Health-related quality of life and fatigue will be assessed.
- Our findings may not be generalisable to other countries, as all participants were recruited in the Netherlands.

## Introduction

 Ulcerative colitis (UC) is a chronic inflammatory bowel disease (IBD) affecting millions of people worldwide, with a continuingly rising incidence.^1^ Symptoms such as abdominal pain and bloody diarrhoea typically begin in adolescence and lead to life-long substantial morbidity with high associated costs.^1 2^ Current available treatments, including immunosuppressants, immunomodulators and expensive biologics, have an efficacy ceiling of around 30% after 1 year^3^ and are accompanied by potential serious side effects.^4^ Thus, the development of more efficacious and safe treatments is paramount.

The pathogenesis of UC is incompletely understood but involves inappropriate activation of the mucosal immune system, likely driven or maintained by the intestinal microbiota.^1^ Accumulating evidence indicates that patients with UC have an altered gut microbiota composition compared with healthy controls.^5 6^ This altered ecosystem can be targeted with several strategies, including faecal microbiota transplantation (FMT). FMT is highly efficacious for treating recurrent *Clostridioides difficile* infections.^7^ Since this reported treatment success, FMT has been investigated for various other indications, including UC.^8^

At the time of setting up this protocol, four RCTs had been published on FMT in patients with UC with active disease.^9^,^12^ The first published RCT, the Transfer of Faeces in Ulcerative Colitis: Restoring Homeostasis (TURN) trial,^9^ was a phase 2a proof-of-concept trial conducted by our group. This trial found no statistically significant difference in clinical and endoscopic remission between patients who received two fresh nasoduodenal donor FMT infusions (30.4%) compared with those who received infusions with autologous FMT (20.0%). However, in-depth microbiota analysis of recipients and donors revealed interesting findings related to clinical outcomes.^9 13^

Response was associated with a microbial composition shift towards the donor’s profile, including increase of alpha-diversity and restoration of *Clostridium* clusters IV, XIVa and XVIII, which belong to the *Firmicutes* (currently renamed to *Bacillota*) phylum.^13^ Sustained remission (⩾1 year) was linked to known butyrate-producing bacteria and overall increased butyrate production capacity, as assessed through functional predictions and targeted quantitative PCR (qPCR).^13^ Butyrate, a short chain fatty acid (SCFA),^14^ is primarily produced by species within the Firmicutes phylum (currently renamed to *Bacillota*)^15^ and is known to downregulate proinflammatory responses in intestinal epithelial cells and to support the gut barrier function.^14^ In contrast, non-response and relapse were associated with increased levels of *Proteobacteria* (currently renamed to *Pseudomonadota*) in patients’ baseline samples and high levels of bacteria that are phylogenetically related to the mucus degrader *Ruminococcus gnavus*^16^ in donors.

Three subsequent published RCTs^10^,^12^ reported a significant treatment effect of FMT ranging between 24 and 32%, compared with placebo rates of 5 to 9%. FMT protocols varied greatly regarding the number of infusions, route of administration, single vs multidonor approach and processing techniques. Microbiota analysis of these trials showed an association of response with several factors, including Clostridium cluster IV, XVII and potential butyrate-producing bacteria,^11 12^ similar to the TURN trial results. Remarkably, a Canadian trial by Moayyedi *et al*,^12^ revealed a highly efficacious donor responsible for seven out of nine responders, suggesting a donor-dependent effect. This donor was particularly rich in *Lachnospiraceae* and *Ruminococcus*.

The majority of the gut microbiota consists of oxygen-sensitive bacteria,^17^ under which butyrate producers. Therefore, regular applied aerobic processing techniques of donor stool diminish the bacterial viability of obligate anaerobes compared with strict anoxic processing, as recently published by our group.^18^ An Australian trial by Costello *et al*^10^ used anoxic processing and reported that the bacteria most strongly associated with clinical response were obligate anaerobes.^10^

In line with current guidelines,^19 20^ we use frozen FMT as clinical efficacy does not appear to be negatively affected by a freeze-thaw cycle,^21^ while using frozen samples offers practical advantages and enhances safety by applying quarantine periods before release of FMT syringes until negative donor rescreening.

We hypothesise that in our follow-up TURN2 trial, we can boost treatment efficacy of FMT in patients with UC with mild to moderate disease by:

1. Applying donor selection based on microbiota profile.
2. Using strict anoxic faecal processing to retain viable oxygen-sensitive bacteria, including many butyrate producers.
3. Combining nasoduodenal and rectal administration with proper bowel cleansing to enhance engraftment.

### Objective

This study aims to investigate the efficacy and safety of FMT augmented by donor selection, anoxic faeces processing and repetitive dual-route administration for the treatment of UC.

### Trial design

The TURN2 trial is a randomised, double-blind, placebo-controlled, multi-centre, parallel-group phase 2 trial with open-label extension option for non-responders in the placebo arm. Eligible patients will be randomised to receive anoxic donor FMT or anoxic autologous FMT in a 1:1 allocation ratio.

## Methods and analysis

### Participants, interventions and outcomes

#### Study setting

Two academic hospitals in the Netherlands are participating. The main study centre is the Amsterdam University Medical Centre (UMC), where all study participants are screened and sigmoidoscopies are performed. All other study visits, including the administration of FMTs, are conducted either at the Amsterdam UMC or at the University Medical Center Groningen (UMCG).

#### Study population

Patients diagnosed with UC with mild to moderate disease activity.

### Eligibility criteria

#### Inclusion criteria patients

- Adults aged ≥18 and <70 years at the time of inclusion.
- Established UC according to the Lennard-Jones criteria^22^ with known involvement of the left colon.
- Partial Mayo score of ≥3.
- Faecal calprotectin >250 µg/g.
- Full Mayo score of 5–9.
- Endoscopic Mayo score of ≥2 in either the rectum or sigmoid on screening sigmoidoscopy.
- No or stable dose of thiopurines and/or mesalamines in the preceding 8 weeks.
- No or stable dose of budesonide in the preceding 2 weeks.
- No prednisone use or prednisone ≤15 mg/day in the preceding 2 weeks with a mandatory taper of 5 mg per week starting from week 4.
- Ability to give informed consent.

#### Main exclusion criteria patients

- Crohn’s disease.
- Condition leading to profound immunosuppression.
- Use of anti-tumour necrosis factor-α treatment, integrin antagonists, Janus kinase inhibitors, ustekinumab (monoclonal antibody) and/or methotrexate in preceding 2 months.
- Use of systemic antibiotics and/or probiotics in preceding 4 weeks.
- Use of topical therapy (5-aminosalicylic acid/mesalamine and/or steroids) in the preceding 2 weeks.
- Positive stool cultures for common enteric pathogens (eg, *Salmonella, Shigella, Yersinia, Campylobacter,* Enteropathogenic *Escherichia coli*).
- Positive *C. difficile* stool test.
- Positive dual faeces test for pathogenic parasites (eg, *Dientamoeba histolytica*, *Giardia lamblia*, *Dientamoeba fragilis* and/or *Blastocystis* spp. In the case of the presence of *Blastocystis* spp only exclusion if many or very many *Blastocystis* spp. are seen microscopically).

The complete list of exclusion criteria is provided in online supplemental file 2.

#### Donors

Potential donors are recruited by advertising among employees of the Amsterdam UMC and preclinical medical students. The screening protocol is based on that of the Netherlands Donor Feces Bank^23^ and is in accordance with the 2019 European clinical consensus report.^24^ It has been updated according to new insights and risks,^25^,^27^ including the COVID-19 pandemic. A detailed description of the donor screening process, donor acceptance rates and feasibility has been published recently.^28^ Eligible donors are asked to donate for a period of a maximum of 12 weeks. Rescreening is conducted after 4 weeks after a donation period to account for window periods to detect certain viruses. Only after successful rescreening are quarantined FMT syringes from that particular donation period are released. Donors are offered a modest amount of money and coffee/lunch vouchers for the hospital café per ten donations. Screening criteria and screening timelines are listed in online supplemental tables 3 and 4 and online supplemental figure 1.

#### Inclusion criteria donors

- Adults aged ≥18 and <55 years at the time of inclusion.
- BMI ≥18.5 and ≤26 kg/m².
- Recruited donors are ranked based on expected beneficial microbiota profile. Arbitrarily, the highest 50% of average-ranked donors are included in the study. The following criteria are used for selection: high alpha diversity (assessed using Faith’s phylogenetic diversity), high predicted butyrate production (based on specific genera abundance, qPCR or inference using Phylogenetic Investigation of Communities by Reconstruction of Unobserved States [PICRUSt]), inverse relative abundance of *Ruminococcus gnavus*, inverse relative abundance of Proteobacteria and overall profiles that resemble successful TURN donors (assessed using Weighted UniFrac).
- Ability to give informed consent.

#### Exclusion criteria donors

Extensive exclusion criteria for faeces donors are listed in online supplemental table 5.

### Interventions

#### Faecal microbiota transplantation

**Figure 1:**
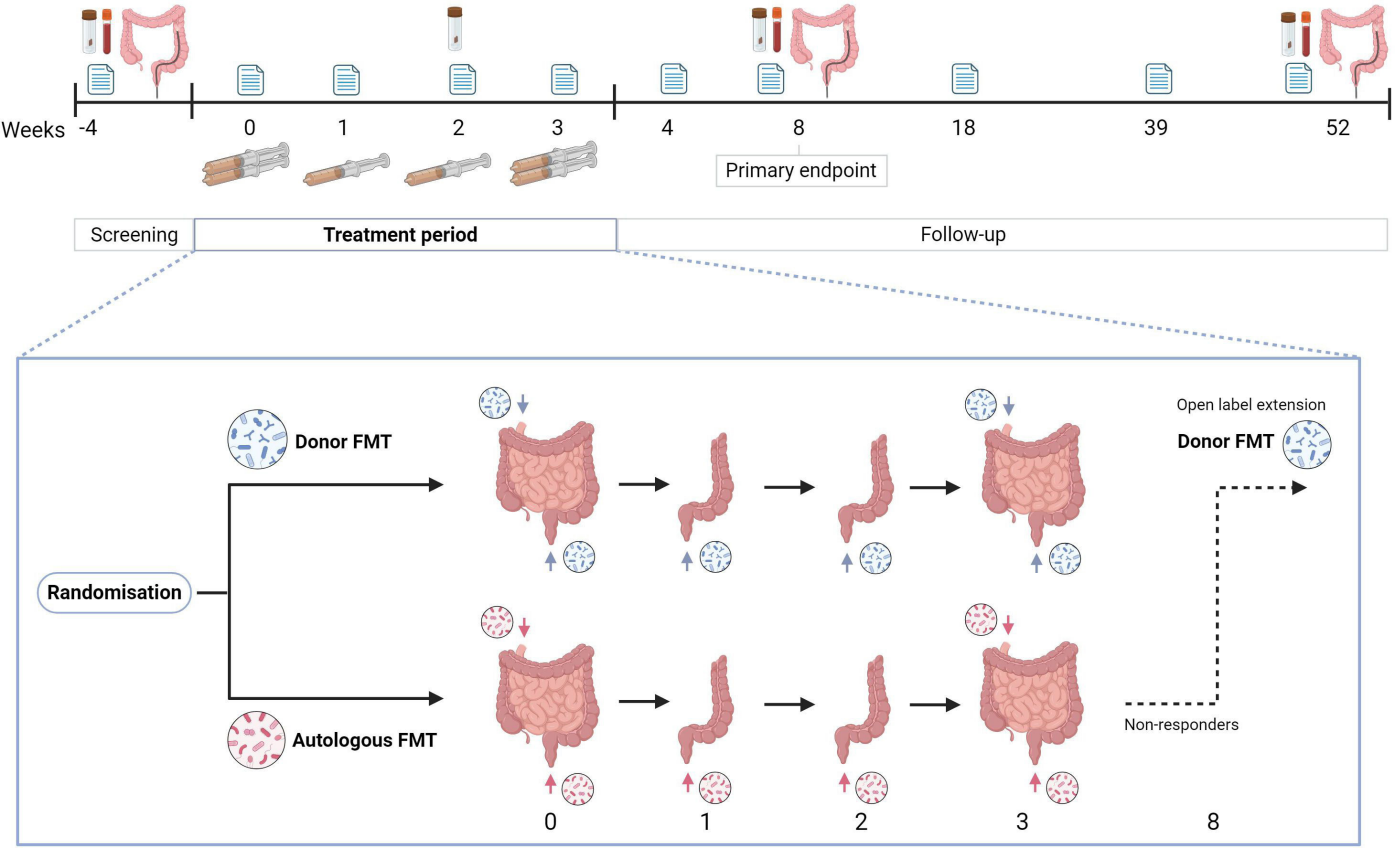
Schematic overview of the study design. FMT, faecal microbiota transplantation.

Patients receive FMT weekly for 4 weeks (figure 1 and online supplemental table 1). Each treatment involves the administration of a 200 mL retention enema. The first and last treatments (week 0 and week 3) consist of dual-route administration, which includes nasoduodenal administration of 300 mL FMT via a CORTRAK* Enteral Access System (Avanos Medical, Alpharetta, USA) and a retention enema. To enhance engraftment, bowel lavage is performed with 3 L macrogol electrolytes solution (Moviprep) beforehand. Patients may choose to either drink the solution or administer it via the CORTRAK tube, which is then placed 1 day before the treatment (visit 2). We added this option to accommodate patients who have difficulty drinking the bowel lavage. In both cases, the bowel lavage is performed at home.

On days when only a retention FMT enema is administered (week 1 and week 2), bowel preparation consists of a sodium chloride (NaCl) enema. NaCl enemas are used instead of phosphate-containing enemas, as the latter can influence the results of microbiota analysis. To increase retention time, loperamide 4 mg is given orally 30 min to 1 hour before FMT administration.

At each FMT visit, a trained nurse or physician is present to provide guidance and reassurance during nasoduodenal placement and/or enema administration. The volume of bowel preparation ingested and the completeness of FMT administration are recorded, and any incomplete intake is documented as a protocol deviation. Patients are encouraged to retain the enema for as long as comfortably possible, and retention time is recorded. Discomfort related to the nasoduodenal tube or rectal administration is assessed at every visit, and symptoms are registered as adverse events (AEs) when applicable.

#### Investigational product

Donor faeces is collected (minimum 20 grams) in an allocated toilet in the Amsterdam UMC in a plastic container placed in a vacuum box (10006101;0 from ALLPAX). Immediately after donation, AnaeroGen sachets (Anaerogen Compact, Thermo Fisher) are added as oxygen scavenger and the container is evacuated to a 97% vacuum. The donation is then transferred to an anaerobic chamber (Anaerobic workstation Concept 500, Baker Ruskin) within 30 min for further processing. This collection and transfer protocol was adjusted from An *et al*,^29^ who demonstrated that their approach facilitated the survival of obligate anaerobes using propidium monoazide (PMA) and culturing. Inside the anaerobic chamber, the stool is weighed and homogenised with NaCl 0.9% in a ratio of 1:3.3–5.5 (depending on consistency) using a blender with a steel autoclavable container (Warring 1LB20ESA). The solution is then filtered using a metal autoclavable tunnel and gauze. Cryoprotectant glycerol (85%) is added to achieve a final concentration of 10%. The prepared FMT is stored in labelled syringes covered with aluminium foil in a −70°C freezer. Before use, syringes are thawed in an anoxic box (AnaeroPack 7.0L, Mitsubishi) with AnaeroGen sachets for either 5 hours at room temperature or overnight in a refrigerator. Patients receive FMT from the same donor for each treatment, while multiple donors are employed throughout the study.

#### Control product

The control product consists of anoxic collected and prepared autologous faeces. This is collected and processed according to the same standard operational procedure. To minimise burden for patients, we collect this stool before the baseline sigmoidoscopy, spontaneously or after patients have received NaCl enema as preparation. Regular laxative enemas containing phosphate were deemed unsuitable due to their potential impact on the microbiota.^30^ The collected stool is diluted with sterile NaCl to a final volume equivalent to that of the investigational product (ie, 1400 mL for four treatments in total).

### Outcomes

#### Primary outcome

The primary endpoint is the proportion of study subjects in steroid-free clinical and endoscopic remission at 8 weeks, assessed using the adapted Mayo score, defined as:

- Stool Frequency Subscore (SFS)≤1
- Rectal Bleeding Subscore (RBS)=0.
- Endoscopic subscore≤1.

The reason for choosing 8 weeks as the primary endpoint was a compromise based on observations from the FATLOSE trial, in which it was observed that the microbiota signature tends to revert to its original composition after 4 weeks,^31^ and from our previous TURN trial, where after twofold nasoduodenal administration of FMT in UC we noticed that several patients responded already within 1 week, while others only after 6 weeks.^9^ Most of the patients who achieved the primary endpoint at 12 weeks were still in clinical remission at 52 weeks.^13^

#### Secondary outcomes

Secondary outcomes include:

- Proportion of patients with:
  - Clinical response per adapted Mayo score at week 8 (ie, excluding Physician’s Global Assessment (PGA) subscore): a decrease from baseline ≥2 points and ≥30%, plus a decrease in RBS≥1 or an absolute RBS≤1.
  - Clinical improvement per partial adapted Mayo score at week 8 (ie, excluding PGA and endoscopy subscore): a decrease from baseline ≥1 points and ≥30%, plus a decrease in RBS≥1 or an absolute RBS≤1.
  - Full clinical remission per full Mayo score at week 8: a decrease from baseline of ≤2 points with no subscore >1.
  - Complete remission per full Mayo score at week 8: ≤2 points reduction from baseline with no subscore >1 and a rectal bleeding score and endoscopic subscore of 0.
  - Sustained steroid-free remission per adapted Mayo at week 8; SFS≤1, RBS=0, endoscopic subscore≤1 and no need for rescue therapy until week 52.
  - Endoscopic complete remission at week 8: endoscopic subscore=0.
  - Endoscopic improvement at week 8: endoscopic subscore of 0 or 1.
  - Endoscopic response at week 8: ≥1 point reduction in summed endoscopic Mayo score (endoscopic subscore of rectum+endoscopic subscore of sigmoid).
  - Histological remission assessed via Nancy Histological Index (NHI)^32^,^34^: NHI≤1.
- Change in:
  - Simple Clinical Colitis Activity Index from baseline to week 1, 2, 3, 4, 8, 18, 39 and 52.
  - Partial Mayo: from baseline to week 1, 2, 3, 4, 8, 18, 39 and 52.
  - IBD-Control: from baseline to week 2, 4, 8, 18, 39 and 52.
- Occurrence of AEs

#### Other explorative outcomes

The gut microbiota composition and functional profiles of donors and recipients in relation to (non-) response will be assessed using shotgun metagenomic sequencing. Strain tracking will be employed to determine donor-derived strain engraftment. Gut microbial-derived metabolites will be analysed using untargeted and targeted approaches, such as high-performance liquid chromatography-mass spectrometry, to measure metabolites involved in cell signalling, with a particular focus on SCFAs and tryptophan. Changes in yeast composition and growth will be assessed through culture techniques. The intestinal immune cell profile in relation to clinical outcomes will be analysed using cytometry by time-of-flight on colon biopsies. Luminex multiplex technology will be performed on supernatants from processed biopsies to assess changes in cytokine and chemokine profiles.^35^ Habitual dietary intake will be obtained by a semiquantitative questionnaire, the Groningen IBD Nutritional Questionnaire (GINQ)^36^ and a 3-day food diary. We will explore associations between the habitual diets of participants (both donors and patients) and clinical outcome. Health-related quality of life will be measured with the SF-36 questionnaire,^37^ and fatigue will be assessed with the Functional Assessment of Chronic Illness Therapy-Fatigue questionnaire.^38^ Other outcomes include changes in the mucosal transcriptome.

### Sample size calculation

At the time of writing the initial protocol, four RCTs^9^,^12^ had been published on FMT for inducing remission in UC, including our previous FMT trial (TURN).^9^ In the TURN trial, the primary endpoint was reached in 30% of patients, with a placebo response rate of 20%.^9^ The other three published RCTs had efficacy rates ranging from 24 to 32% and placebo rates from 5% to 9%.^9^,^12^ In the current study, we aim to augment the treatment effect by selecting faeces donors on their microbiota profile, using anoxic processing, and by promoting engraftment through dual-route administration and repeated FMTs. We therewith estimate to boost the treatment effect to 33–40%. By using a more stringent endpoint, we anticipate a lower placebo compared with the TURN trial, aligning with rates observed in other studies.

The sample size calculation was performed using nQuery software (Cork, Ireland) with the following assumptions: efficacy in the placebo group of 10%, efficacy in the treatment group of 37%, two-sided significance level (α) of 0.05 and desired power of 80% (β=0.20). Based on these parameters, a sample size of 38 patients per group, totalling 76 patients, is required. The study incorporates an adaptive design that allows for an increase in the sample size up to a maximum of 100 patients to account for (early) drop-outs (online supplemental table 6).

### Recruitment

Patients are recruited from the outpatient clinics of the Amsterdam UMC and UMCG by their treating gastroenterologist. Additionally, patients from other hospitals in the Netherlands can be referred to the Amsterdam UMC by their treating gastroenterologist or general practitioner. To inform patients and healthcare practitioners about the trial, recruitment efforts include advertisements on the Amsterdam UMC intranet webpage, a general webpage and Google Ads, a dedicated Facebook page and through the Dutch Maag Lever Darm Stichting.

### Patient allocation and blinding

Randomisation is performed using a computerised random-number generator within the Electronic Data Capture system Castor, allocating patients in a 1:1 ratio to either allogenic (donor) FMT or autologous FMT. Randomly permuted blocks of 2, 4 or 6 are applied with no additional stratification. A research assistant conducts the randomisation and subsequently prepares autologous FMT or collects syringes with donor FMT (based on Epstein-Barr virus and Cytomegalovirus matching), according to the allocation outcome.

To ensure blinding, FMT syringes are visually and olfactorily concealed in capped syringes covered with aluminium foil and labelled identically for autologous and donor FMT. All labels include a tearable section with the allocation and a unique code. A second independent research assistant verifies either the preparation of autologous FMT or the correct matching of donor syringes and removes the tearable labels, which are maintained in a drug accountability log. The research assistants are the only individuals who are unblinded and have no further role during the study. FMT administration and all visits are conducted by a blinded research nurse or fellow. Gastroenterologists performing sigmoidoscopies and treating physicians are also blinded.

De-blinding of investigators may occur after all patients have reached the primary endpoint (week 8), to facilitate data publication. Patients who do not meet the secondary endpoint of clinical response at week 8 and who have an endoscopic Mayo subscore of ≥2 at week 8 can be unblinded if they wish to continue with open-label extension. Open-label extension with donor FMT will only be available to patients who were originally allocated to autologous FMT.

### Data collection

The assessments are summarised in online supplemental table 1 and a schematic overview of the interventions and assessments per timepoint is provided in figure 1. Patient data are handled with strict confidentiality and recorded in a pseudonymised format using unique participant codes. The code lists (ie, subject identification log) are digitally stored on a secure server hosted by the Amsterdam UMC and are only accessible to the principal investigator (PI) and project leaders. Data are saved in a secure online database (Castor, CIWIT BV, Amsterdam). All source documents and case report forms (CRFs) will be stored at the Amsterdam UMC for 15 years following the trial’s closure. All personnel involved in data entry receive appropriate training.

For patients who drop out of the study due to safety concerns (eg, worsening of disease), adjusted follow-up visits at similar timepoints (V10, T39, V11) will be conducted to collect safety data, including AEs and safety blood analysis at V11. If patients choose to withdraw from the study, no further follow-up data will be collected.

Videotaped sigmoidoscopies will be evaluated by a critical event committee (CEC), composed of two experienced IBD gastroenterologists, who will be blinded for randomisation allocation and the sequence of videos. Scoring will be conducted individually. In case of unequal scoring, videos will be reviewed together and discussed until consensus is reached.

### Statistical methods

Primary and secondary outcomes will be analysed using a ‘modified intention-to-treat’ design, meaning that data from all randomised patients will be analysed, with the exception of data of those who did not receive any FMT treatment (ie, patients who dropped out before V3). For the primary endpoint, the analysis will use a conservative approach in which participants with missing week 8 outcome data are classified as not in remission, irrespective of reason for missingness. To correct for missing data due to drop-outs, additional randomisation will be performed according to a fixed schedule outlined in online supplemental table 6.

Demographic data will be reported as either the mean with a 95% CI, or as the median and IQR for data not normally distributed. Analysis of the primary endpoint will be will be calculated using a two-sided χ^2^ test. Descriptive and exploratory data analysis will be reported as per the described primary and secondary endpoints. Statistical significance will be established at p<0.05 with a CI of 95%. P values of secondary outcomes will be adjusted using the Holm-Bonferroni procedure to account for multiple testing. Statistical analyses will be conducted using SPSS V.29.0 software (Chicago, IL) or R programming language version 4.

To identify dietary patterns assessed with the GINQ, principal component analyses will be performed on 22 food groups. Flare occurrence after FMT will be analysed using multivariable Cox proportional hazards models. To identify microbial candidates that are associated with response to FMT, we will perform differential abundance analysis comparing responders versus non-responders using linear regression analyses considering age, sex, BMI, proton pump inhibitor use, antibiotic and sequencing read depth as covariates. Next, we will analyse data derived from the longitudinal monitoring to detect changes after FMT and donor-derived engraftment of species associated with (non)-response.

### Monitoring

The risk associated with this study is assessed as ‘moderate’ according to the guideline on quality assurance in human research provided by the Dutch Federation of University Medical Centers. Consequently, monitoring is required and is conducted by the Clinical Research Unit of the Amsterdam UMC. This data monitoring is independent from the sponsor and any competing interests. Five monitoring visits are scheduled throughout the study, following a detailed monitoring plan to ensure the protection of the participant’s rights and the accuracy of data. Furthermore, a monitor verifies the compliance of the study with applicable laws and regulatory requirements (eg, the Medical Research Involving Human Subjects Act (WMO), International Council for Harmonisation of Technical Requirements for Pharmaceuticals for Human Use-Good Clinical Practice (ICH-GCP) guidelines and/or ISO14155). After every visit, a report will be generated and shared with the head of the department and coordinating PI. Any issues identified must be resolved within 4 weeks for high-priority concerns and 10 weeks for low-priority concerns.

In addition to regular monitoring, we established an independent Data Safety Monitoring Board (DSMB) to perform interim analysis for efficacy and safety surveillance. Patient data will be disclosed to the DSMB when half of the intended sample size (N=38) is evaluable for the primary endpoint. The advice of the DSMB following the interim analysis will be shared with the PI and the Institutional Review Board (IRB).

The following stopping guidelines were defined:

1. Unacceptable occurrence of serious adverse events (SAEs) as judged by the DSMB members in conjunction with the IRB.
2. Futility, defined as:
  - A difference in treatment effect of ≤5% in favour of the active arm. In this case, the DSMB may advise the study team to extend the sample size, allowing the study team to decide whether to continue or terminate the trial.
  - A difference in treatment effect of >5% in favour of the placebo group. In this case, the trial will be discontinued.

### Harms

AEs will be monitored throughout the study. All AEs reported (spontaneously) by the patient, observed by the study team or communicated by other healthcare practitioners will be recorded. SAEs, as defined by the Centrale Commissie Mensgebonden Onderzoek (CCMO), are reported to the IRB within 15 days (or within 7 days if life-threatening). If SAEs are considered directly related to the FMT treatment, the DMSB will be notified within 24 hours. All AEs will be tracked until resolution or stabilisation. Follow-up of AEs may require additional tests, medical procedures and/or referrals to a general physician or medical specialist.

This study involves sigmoidoscopies with tissue sampling, CORTRAK placements and FMTs. The risks associated with these procedures are cited in the written patient information. Sigmoidoscopies with biopsies carry a small risk of complications, including bleeding and perforation.^39^ Feeding tube placements, including those with nasoduodenal tube positioning through a CORTRAK electromagnetic sensing device, involve a low risk of complications including aspiration, malpositioning and perforation.^40 41^ If there is any suspicion of tube malposition, a plain thoracic and/or abdominal X-ray will be performed. To prevent complications, sedation around the CORTRAK and FMT procedures is not allowed and patients with swallowing disorders will not be included in this study.

Prior studies have concluded that FMT is generally a safe therapy, with mostly transient AEs such as transient bloating, flatulence, abdominal pain and increase of stool frequency.^42^ SAEs are rare.^42^ Strict regulations apply for donor screening, minimising the risk of pathogen transmission (details on screening are listed in online supplemental tables 3 and 4 and online supplemental figure 1).

### Ethics and dissemination

Ethics approval was obtained from the IRB of the Amsterdam UMC in the Netherlands (reference number 2018_057), in accordance with the principles of the Declaration of Helsinki (version 2013) and in compliance with the WMO. The trial was prospectively registered on 25 May 2018 in the Dutch Trial Register (NTR/LTR) as NL7770 and was re-assigned the identifier NL-OMON52507 following the transition of the Dutch Trial Register to the Overview of Medical Research in the Netherlands (OMON). The trial is also registered at ClinicalTrials.gov (NCT05998213). All participants will provide written informed consent, which can only be obtained by the trial coordinators and the trained trial nurse. An example of the participant informed consent form is attached in the online supplemental materials. Any proposals for protocol amendments will be submitted to the IRB for approval prior to implementation. Relevant changes affecting participants will be communicated by letter, including an additional informed consent form. Patients can indicate whether they approve the use of their data and biological specimens beyond 5 years after closure of the trial and whether they are willing to be approached for follow-up studies. The results of the trial will be disseminated through publication in a peer-reviewed journal and presentations at (inter)national conferences.

### Patient and public involvement statement

Patients or the public were not involved in the design, or conduct, or reporting or dissemination plans of our research.

## Discussion

The TURN2 trial is a follow-up study incorporating lessons learnt from the TURN trial^9 13^ with the aim to increase the efficacy of FMT in patients with UC. In this study, we select faeces donors based on their microbiota profiles and apply strict anoxic processing to retain the viability of beneficial microbes. We further use dual-route administration with prior bowel cleansing to enhance engraftment. We hypothesise that these measures will improve the efficacy reported in previous studies.

As of July 2024, full reports of six double-blind RCTs have been published on FMT in UC adults,^9^,^1243 44^ pilot studies not included. Meta-analyses indicate that FMT is superior to placebo in inducing remission for patients with active disease.^45 46^ However, substantial heterogeneity among trial protocols^47 48^ complicates the generalisation of these results.

The mechanism of action of FMT in UC is as yet unclear, but it likely involves a mediating effect of live bacteria and their metabolites. Given that the majority of gut microbiota are anoxic in nature,^17^ preserving these bacteria by minimising their exposure to oxygen might be crucial for their functionality in the new host. Consequently, anoxic processing of donor stool may be an important factor in improving clinical outcomes. However, to date, only two double-blind RTCs have used anaerobic processing. In the Australian trial by Costello *et al*,^10^ anoxic prepared donor FMTs had a significant effect in inducing remission compared with aerobically prepared autologous FMT. Conversely, a recent trial that used anoxic stool processing for both treatment arms was halted for futility.^43^ The authors of the latter trial attributed the lack of effect to several factors, including high disease severity, a long average disease duration and high pre-exposure to biological treatments in their cohort.

We designed a highly stringent anoxic protocol which involves immediate collection of donor stool in vacuum boxes with oxygen scavengers. The samples are then swiftly transferred to an anaerobic chamber within 30 min after donation for further processing. Recently, we demonstrated that this anoxic working protocol retains potentially beneficial strains^18 49^ compared with aerobic processing.^18^ Although our optimised protocol is translatable to clinical practice, the use of a specialised infrastructure may hamper generalisability.

A donor-dependent effect has been reported in several clinical trials.^11 12 44 50^ In the current study, we select donors based on pre-defined microbial criteria that associate with clinical outcomes, as identified in the TURN trial and other studies. These factors include high predicted butyrate production and high alpha diversity, the latter being one of the most consistent findings reported in literature.^49 51^

Creating a reliable placebo for FMT is challenging. For blinding purposes, autologous FMT serves as a safer comparator arm than saline or water. However, the TURN trial^9^ showed a high ‘treatment’ effect with this kind of ‘placebo’ treatment, potentially complicating the ability to demonstrate a true effect size of donor FMT. Although it is unclear if and how autologous FMT would induce a response in UC, we aim to lower the placebo effect in the current study by applying a stricter primary endpoint. This endpoint includes both endoscopic and clinical parameters, incorporated in the Mayo score. To enhance the reliability of the primary endpoint, we have established a blinded CEC consisting of two experienced gastro-enterologists who independently evaluate videotaped sigmoidoscopies.

Recruitment difficulties are a frequent issue in RCTs.^52^ The COVID-19 pandemic, which occurred during the recruitment phase of the trial (September 2019 to present), likely affected both donor and patient recruitment. To enhance recruitment, we successfully used broader advertisement strategies, including social media and a website with paid Google Ads, following the positive experience of another study within our department.^53^

As recently elaborated,^28^ faeces donor screening involves significant financial burdens and practical challenges. During the conduct of our research, our faeces donor screening protocol had to be adjusted multiple times in response to new risks (ie, the COVID-19 pandemic^26^ and the monkeypox outbreak^27^ and new insights ie, stricter guidelines on Multidrug-Resistant Organism (MDRO) screening following reported SAEs to the US Food and Drug Administration (FDA)).^25^ These updated guidelines made it even more difficult to find suitable donors. We recruit faeces donors among the hospital staff and medical students to facilitate rapid anoxic processing after donation on site. However, after the FDA’s 2019 warning on MDRO risk, we could no longer recruit healthcare workers with patient contact, resulting in a smaller screening cohort. Even when a suitable faeces donor is identified, retaining them over extended periods can be difficult.

Despite the promising outcomes of FMT for patients with UC, these challenges and the inability to standardise FMT make its use in daily practice, in current format, unlikely. Nevertheless, further research is critically needed to address the many remaining questions around effective FMT protocols and to allow future simplification into more practical and standardisable FMT therapy for clinical use. Understanding the mechanisms of action is imperative for advancing the field and developing effective, practical, microbial therapies. These questions are addressed within this protocol via the investigation of additional microbial and metabolomic analyses. By combining microbiome, metabolome, mucosal immune and dietary data, TURN2 is designed not only to test an optimised FMT protocol but also to identify predictive biomarkers that could ultimately enable personalised donor-recipient matching or the development of defined microbial therapeutics.

The TURN2 study aims to provide evidence for the use of anoxic processed, microbial selected donor FMT in patients with active UC. It seeks to add substantial knowledge into specific strains, metabolites and microbial functions that are related to clinical FMT response.

### Trial status

Recruitment of faeces donors started in December 2018. The first study patient was included in June 2020. Recruitment was completed in November 2024, and the follow-up period will continue until January 2026.

## Supplementary material

10.1136/bmjopen-2025-107097 online supplemental file 1

10.1136/bmjopen-2025-107097 online supplemental file 2
