# Supplementary material for: Transfer of faeces in ulcerative colitis 2: improving efficacy – study protocol for a multicentre randomised controlled trial (TURN2 study)
Source: BMJ Open. 2026 May 5;16(5):e107097. doi: 10.1136/bmjopen-2025-107097 (PMC13150868; doi:10.1136/bmjopen-2025-107097)
Supplement: online supplemental file 1 [file bmjopen-16-5-s001.docx]

**Supplemental Table 1. Participant timeline**

|  | **STUDY PERIOD** | | | | | | | | | | | | | |
| --- | --- | --- | --- | --- | --- | --- | --- | --- | --- | --- | --- | --- | --- | --- |
|  | **Enrolment** | **Allocation** | **Treatment phase and primary endpoint (PE)** | | | | | | | | **Open-label extension^1^** | **Follow-up** | | |
| **VISIT** | **V1** |  | **V2** | **V3** | **V4** | **V5** | **V6** | **V7** | **V8** | **V9** | **OLE V2 – OLE V9** | **V10**^2^ | **t39^3^** | **V11** |
| **WEEK** | **-4 to 0** |  | **0^-1^** | **0** | **1** | **2** | **3^-1^** | **3** | **4** | **8** | **9 - 17** | **18** | **39 (48^4^)** | **52 (61^4^)** |
|  |  |  |  | **FMT 1** | **FMT 2** | **FMT 3** |  | **FMT 4** |  | **PE** |  |  |  |  |
| **ENROLMENT:** |  |  |  |  |  |  |  |  |  |  |  |  |  |  |
| **Eligibility screen** | X |  |  |  |  |  |  |  |  |  |  |  |  |  |
| **Informed consent** | X |  |  |  |  |  |  |  |  |  |  |  |  |  |
| **Allocation** |  | X |  |  |  |  |  |  |  |  |  |  |  |  |
| **INTERVENTIONS:** |  |  |  |  |  |  |  |  |  |  |  |  |  |  |
| ***Allogenic FMT*** |  |  |  |  |  |  |  |  |  |  |  |  |  |  |
| ***Autologous FMT*** |  |  |  |  |  |  |  |  |  |  |  |  |  |  |
| ***CORTRAK placement*** |  |  | X |  |  |  | X |  |  |  | X |  |  |  |
| ***FMT enema*** |  |  |  | X | X | X |  | X |  |  | X |  |  |  |
| ***FMT nasoduodenal*** |  |  |  | X |  |  |  | X |  |  | X |  |  |  |
| **ASSESSMENTS:** |  |  |  |  |  |  |  |  |  |  |  |  |  |  |
| ***Sigmoidoscopy + biopsies*** | X |  |  |  |  |  |  |  |  | X | X |  |  | X |
| ***Fecal calprotectin*** | X |  |  |  |  |  |  |  |  | X | X |  |  | X |
| ***Feces analysis (screening)*** | X |  |  |  |  |  |  |  |  |  | X |  |  |  |
| ***Blood analysis (screening and safety)*** | X |  |  |  |  |  |  |  |  | X | X |  |  | X |
| ***Storage feces sample*** | X |  |  |  |  | X |  |  |  | X | X |  |  | X |
| ***Storage blood sample*** | X |  |  |  |  |  |  |  |  | X | X |  |  | X |
| ***SCCAI, partial Mayo*** | X |  | X |  | X | X | X |  | X | X | X | X | X | X |
| ***IBD-control*** | X |  | X |  |  | X |  |  | X | X | X | X | X | X |
| ***SF-36, FACIT-F*** | X |  | X |  |  |  |  |  | X | X | X | X |  | X |
| ***GINQ*** | X |  |  |  |  |  |  |  |  |  |  |  |  |  |
| ***Food diary*** | X |  |  |  |  |  |  |  |  |  |  |  |  | X |
| ***Change in medication*** | X | X | X | X | X | X | X | X | X | X | X | X | X | X |
| ***Adverse events*** | X | X | X | X | X | X | X | X | X | X | X | X | X | X |

^1^ All study assessments and interventions during the open-label extension are identical to the initial treatment phase. For specifications during this period, see the columns ‘treatment phase and primary endpoint’. The primary endpoint of the initial phase serves as rescreening and starting point of the open-label extension.

^2^ Visit 10 decades for the patients in open-label extension.

^3^ t39 is a phone call instead of a physical visit.

^4^ For the open-label extension patients follow up is conducted at the same intervals after the first treatment (+39 weeks, +52 weeks) starting from the first FMT in open-label extension (week 9).

**Supplemental Table 2:** **Exclusion criteria patients**

| **Symptoms and comorbidity** |
| --- |
| Crohn’s disease; |
| Condition leading to profound immunosuppression (e.g., HIV, bone marrow malignancies, use of systemic chemotherapy, Child-Pugh B liver cirrhosis) |
| Life expectancy < 12 months; |
| History of surgery: presence of a pouch or stoma; |
| Known intra-abdominal fistula; |
| Signs of ileus, diminished passage; |
| Difficulty with swallowing; |
| Pregnancy, breast-feeding, or no use of reliable anticonception in fertile woman; |
| **Medicine and allergies** |
| Use of anti-TNFα treatment, vedolizumab, tofacinitib, ustekinumab, methotrexate in preceding 2 months; |
| Use of cyclosporine in preceding 4 weeks; |
| Use of systemic antibiotics and/or probiotics in preceding 4 weeks; |
| Use of topical therapy (5-ASA and/or steroids) in preceding 2 weeks; |
| Use of prednisone dose >15 mg/day in preceding 2 weeks; |
| Vasopressive medication, intensive care unit stay; |
| Allergy to macrogol or substituents; |
| **Tests** |
| Positive *Clostridioides difficile* stool test; |
| Positive stool cultures for common enteric pathogens (e.g., *Salmonella, Shigella, Yersinia, Campylobacter, Enteropathogenic E. coli*); |
| Positive dual feces test for pathogenic parasites (e.g., *Dientamoeba histolytica*, *Giardia Lamblia*, *Dientamoeba fragilis*, and/or *Blastocystis spp*. In the case of presence of *Blastocystis* spp. only exclusion if microscopically many or very many *Blastocystis* spp. are seen); |
| Positive serological test for HIV; |
| **Other** |
| Subject who has any conditions that in the opinion of the investigator, would compromise the safety of the subject or the quality of the data and is an unsuitable candidate for the study. |

Abbreviations: TNF; tumor necrosis factor, 5-ASA; mesalamines, 5-aminosalicylate, HIV; human immunodeficiency viruses.

**Supplemental Table 3:** **Specification of donor screening**

| **Feces screening** | |
| --- | --- |
| ***Bacteria*** *(PCR or stool antigen detection^a^)* | |
| *Aeromonas spp.* | *Salmonella spp.* |
| *Clostridium difficile* | Shiga toxin-producing *Escherichia coli* (STEC) |
| *Helicobacter pylori* | *Shigella spp*. |
| Pathogenic *Campylobacter spp*. | *Yersinia enterocolitica* |
| *Plesiomonas shigelloides* |  |
| ***Multidrug resistant organisms*** *(culture)* | |
| Carbapenem-resistant *Enterobacteriaceae* (CRE) | Multidrug-resistant Gram-negatives (MRGN) 3 |
| ESBL-producing *Enterobactereacceae* | MRGN 4 |
| Methicillin-resistant *Staphylococcus aureus* (MRSA) | Vancomycin-resistant *Enterococcus* (VRE) |
| ***Viruses*** *(PCR)* | |
| Adenovirus non-41/41 | Norovirus Type I and II |
| Adenovirus type 40/41 | Parechovirus |
| Astrovirus | Rotavirus |
| Enterovirus | Sapovirus |
| Hepatitis E virus | Severe acute respiratory syndrome coronavirus 2 |
| ***Parasites*** *(PCR and/or microscopic evaluation)* | |
| *Blastocystis* spp*.^b^* | *Entamoeba moshkovskii* *^c^* |
| *Cryptosporidium* spp. | *Entamoeba polecki ^c^* |
| *Cyclospora* | *Giardia lamblia* |
| *Dientamoeba fragilis* | *Iodamoeba bütschlii* *^c^* |
| *Endolimax nana^c^* | *Isospora spp*. |
| *Entamoeba coli* *^c^* | Larvae ^c^ |
| *Entamoeba dispar* *^c^* | *Microsporidium spp*. |
| *Entamoeba gingivalis* *^c^* | Parasitic worm eggs ^c^ |
| *Entamoeba hartmanni* *^c^* | Protozoan Cysts and Oocysts ^c^ |
| *Entamoeba histolytica* |  |
| **Serum screening** | |
| ***Bacteria*** *(ELISA)* | |
| *Treponema pallidum* |  |
| ***Viruses^d^*** *(CLIA or PCR)* | |
| *Cytomegalovirus* (CMV)^d^ | *Hepatitis C virus*^d^ |
| *Epstein-Barr Virus* (EBV) | *Human immunodeficiency viruses* (HIV)^d^ |
| *Hepatitis B virus*^d^ | *Human T-lymphotropic virus Type I and II (HTLV)* |
| ***Parasites*** *(ELISA)* | |
| *Strongyloides stercoralis* |  |

^a^ All bacteria are detected with the use of PCR, with exception of Helicobacter pylori were ELISA is used

^b^ Exclusion of donor only if high amounts Blastocystis spp. are seen with microscopy, defined as ‘moderate’ or ‘many’ (REF Garcia)

^c^ Presence of only one non-pathogenic parasite is acceptable

^d^ Initial screening for these viruses is performed using serology, while rescreening is performed with PCR

**Supplemental Table 4:** **Time intervals of donor screening**

|  | **Donor initial screening** | **Donation period** *(max. 12 weeks)* | **60-day screening** (*60 days after start donation period)* | **Full rescreening**  *(+ 4 weeks after end donation period)* |
| --- | --- | --- | --- | --- |
| **Inclusion and exclusion criteria** | X |  |  |  |
| **Informed consent** | X |  |  |  |
| **Screening questionnaire** | X |  |  |  |
| **Parasitology - stool** | X |  |  | X |
| **Virology - stool** | X |  |  | X |
| **Bacteriology - stool** | X |  |  | X |
| **MDROs – stool** | X |  | X | X |
| **SARS-CoV-2 – stool** | X |  | X |  |
| **Blood screening serology** | X |  |  | X |
| **Blood screening serology + PCR** |  |  |  | X |
| **Microbiota profiling** | X |  |  |  |
| **GINQ** | X |  |  |  |
| **Anoxic stool collection** |  | X |  |  |

**Supplemental Figure 1:** **Timelines donor screening**

**Figure 1a. Simplified overview of donor screening, example with one donation period.**

**Figure 1b. Simplified overview of donor screening, example with multiple donation periods.**

**Supplemental Table 5:** **Exclusion criteria for feces donors**

| **Symptoms and comorbidity** |
| --- |
| Abnormal bowel motions, abdominal complaints, or symptoms indicative of irritable bowel syndrome |
| Symptoms indicative of monkeypox and/or SARS-CoV-2 |
| Inflammatory bowel disease |
| Active hepatitis B, active hepatitis C, acute infection with CMV or EBV |
| Risk of Creutzfeld-Jacob disease |
| Untreated infection with: Treponematose, TB, and/or Herpes virus |
| History or clinical evidence of autoimmune disease (e.g., type 1 diabetes, Hashimoto’s hypothyroidism, Graves’ hyperthyroidism, rheumatoid arthritis, celiac disease) |
| Presence or history of psychiatric disease (e.g., depression, schizophrenia, autism, Asperger’s syndrome) |
| Presence or history of chronic neurological/neurodegenerative disease (e.g., Parkinson’s disease, multiple sclerosis) |
| **Behavioral criteria** |
| Extensive travel behavior; |
| Higher risk of colonization with multidrug-resistant organisms, including:   - Health care workers with direct patient contact - Persons who have recently been hospitalized or discharged from long-term care facilities - Persons who regularly attend outpatient medical or surgical clinics - Persons who have recently engaged in medical tourism |
| Predisposing factors for potential transmittable diseases (e.g., unsafe sex practice, regular sexual contact with prostitutes, illicit drug use) |
| Smoking |
| **Medicine** |
| Use of antibiotics in the past 4 weeks |
| Use of any medication except oral contraceptives and over-the-counter incidental medication |
| History of treatment with growth factors |
| **Positive tests** |
| Positive blood test for the presence of HIV, HTLV-I and HTLV-II, *Strongyloides*, lues |
| Positive feces test for bacteria: *Campylobacter, C. difficile*, *Salmonella*, *Shigella*, *Yersinia*, enteropathogenic *E. coli*, *Helicobacter pylori*, *Aeromonas* spp., *Plesiomonas shigelloides*. |
| Presence of parasites (assessed with Dual Feces Test): *Giardia lamblia*, *Dientamoeba fragilis*, *Entamoeba histolytica*, *Microsporidia*, *Cryptosporidium* antigen, *Cyclospora* and *Isospora*, *Blastocystis* spp. only of microscopically many or very many blastocysts are seen, *Strongyloides*, microscopic positive exam for eggs, cysts and larves (e.g., helminth eggs). |
| Positive feces test for: *Rotavirus*, *Norovirus*, *Enterovirus*, *Parechovirus*, *Astrovirus*, *Sapovirus*, Adenovirus 40/41 and non 40/41, hepatitis E, SARS-CoV-2. |
| Positive feces test for antibiotic resistant bacteria: ESBL, CRE, VRE, MRSA. |

Abbreviations: SARS-CoV-2, severe acute respiratory syndrome coronavirus; CMV, *Cytomegalovirus*; EBV, Epstein-Barr virus; TB, tuberculosis; HIV, human immunodeficiency viruses; HTLV-I and HTLV-II, human T-lymphotropic virus type I and II; ESBL, extended spectrum beta-lactamase; CRE, carbapenem-resistant *Enterobacteriaceae*; VRE, Vancomycin-resistant *Enterococcus*; Methicillin-resistant *Staphylococcus aureus* (MRSA).

**Supplemental Table 6:** **Additional randomisation**

| **Situation** | **Timepoint study** | **Secondary endpoint**  **t= 4 weeks**  **(SCCAI)** | **Primary endpoint**  **t= 8 weeks** | **Action on total sample size** |
| --- | --- | --- | --- | --- |
| 1.A Start additional medicinal treatment  Because of ‘ongoing symptoms’ | t < 4 weeks | Not evaluable  Score as therapy failure | Not evaluable  Score as therapy failure  *Non-responder imputation (NRI)* | Randomisation:  + 1 extra |
| 1.B Start additional medicinal treatment  Because of ‘ongoing symptoms’ | t > 4 weeks | Evaluable | Not evaluable  Score as therapy failure  *NRI* | - |
| 2.A Study termination:  Reason not disease related | t < 4 weeks | Score as therapy failure | Not evaluable  *NRI* | Randomisation:  + 1 extra |
| 2.B Study termination:  Reason not disease related | t > 4 weeks | Evaluable | V8 data (week 4) are used as primary endpoint data (V9, week 8)  *Last observation carried forward* | - |
