# Supplementary material for: Transfer of faeces in ulcerative colitis 2: improving efficacy – study protocol for a multicentre randomised controlled trial (TURN2 study)
Source: BMJ Open. 2026 May 5;16(5):e107097. doi: 10.1136/bmjopen-2025-107097 (PMC13150868; doi:10.1136/bmjopen-2025-107097)
Supplement: online supplemental file 2 [file bmjopen-16-5-s002.pdf]

Bijlage C: Toestemmingsformulier proefpersoon

Feces transplantatie met ontlasting van een gezonde donor bij patiënten met colitis ulcerosa, de TURN 2 trial.

- Ik heb de informatiebrief gelezen. Ook kon ik vragen stellen. Mijn vragen zijn voldoende beantwoord. Ik had genoeg tijd om te beslissen of ik meedoe.
- Ik weet dat meedoen vrijwillig is. Ook weet ik dat ik op ieder moment kan beslissen om toch niet mee te doen of te stoppen met het onderzoek. Daarvoor hoef ik geen reden te geven.
- Ik geef toestemming voor het informeren van mijn huisarts en specialist die mij behandelt dat ik meedoe aan dit onderzoek.
- Ik weet dat voor de controle van het onderzoek sommige mensen mijn gegevens kunnen inzien. Die mensen staan vermeld in deze informatiebrief. Ik geef toestemming voor die inzage.
- Ik geef toestemming voor het verzamelen en gebruiken van mijn gegevens/bloedmonsters/lichaamsmateriaal op de manier en voor de doelen die in de informatiebrief staan.
- Ik geef toestemming om mijn gegevens gecodeerd nog maximaal 15 jaar na afloop van dit onderzoek te bewaren.
- Ik weet dat ik niet zwanger mag worden tijdens het onderzoek.
- Ik geef
  □ **wel**
  □ **geen**
  toestemming om mijn lichaamsmateriaal nog 5 jaar na dit onderzoek te bewaren. Mogelijk kan dit later nog voor meer onderzoek worden gebruikt, zoals in de informatiebrief staat.
- Ik geef
  □ **wel**
  □ **geen**
  toestemming om mij na dit onderzoek opnieuw te benaderen voor een vervolgonderzoek
- Ik wil
  □ **wel**
  □ **niet**
  geïnformeerd worden over welke behandeling ik heb gehad/in welke groep ik zat.
- Ik wil meedoen aan dit onderzoek.

Naam proefpersoon:
Handtekening: Datum : __ / __ / __

Ik verklaar dat ik deze proefpersoon volledig heb geïnformeerd over het genoemde onderzoek.
Als er tijdens het onderzoek informatie bekend wordt die de toestemming van de proefpersoon zou kunnen beïnvloeden, dan breng ik hem/haar daarvan tijdig op de hoogte.
Naam onderzoeker:
Handtekening: Datum: __ / __ / __

Appendix C: Participant consent form (translated)

Faecal transplantation with stool from a healthy donor in patients with ulcerative colitis, the TURN 2 trial.

- I have read the information letter. I was also able to ask questions. My questions have been answered satisfactorily. I had enough time to decide whether I want to participate.
- I understand that participation is voluntary. I also understand that I can decide at any moment not to participate after all or to withdraw from the study. I do not have to give a reason for this.
- I give permission to inform my general practitioner and the specialist who is treating me that I am participating in this study.
- I understand that, for the purpose of monitoring this study, certain people may inspect my data. These people are listed in the information letter. I give permission for this access.
- I give permission for the collection and use of my data/blood samples/body material in the manner and for the purposes described in the information letter.
- I give permission for my data to be stored in coded form for up to 15 years after the end of this study.
- I understand that I must not become pregnant during the study.
- I give
  □ yes
  □ no
- permission for my body material to be stored for 5 years after this study. These samples may possibly be used for additional research in the future, as described in the information letter.
- I give
  □ yes
  □ no
- permission to be contacted again after this study for a follow-up study.
- I would like
  □ yes
  □ no
- to be informed about which treatment I received / which group I was in.
- I would like to participate in this study.

Name of participant:
Signature: Date: __ / __ / __

I declare that I have fully informed this participant about the study mentioned above.
If, during the course of the study, information becomes available that could affect the participant's willingness to continue, I will inform him/her of this in good time.
Name of investigator:
Signature: Date: __ / __ / __
