# Supplementary material for: Transfer of faeces in ulcerative colitis 2: improving efficacy – study protocol for a multicentre randomised controlled trial (TURN2 study)
Source: BMJ Open. 2026 May 5;16(5):e107097. doi: 10.1136/bmjopen-2025-107097 (PMC13150868; doi:10.1136/bmjopen-2025-107097)
Supplement: Supplementary file 3 [file bmjopen-2025-107097.reviewer_comments.pdf]

PEER REVIEW HISTORY

BMJ Open publishes all reviews undertaken for accepted manuscripts. Reviewers are asked to complete a checklist review form and are provided with free text boxes to elaborate on their assessment. These free text comments are reproduced below.

ARTICLE DETAILS

Title (Provisional)

Transfer Of Feces In Ulcerative Colitis 2; Improving Efficacy: Study protocol For A Multicenter Randomized Controlled Trial (TURN2 study) Authors

Authors

Bénard, Mèlanie; Van Der Spek, | M.J.W.; Davids, | M; Visser, | C.E.; Zoetendal, | E.G.; Rethans, | B; Zwezerijnen-Jiwa, | F.H.; Visschedijk, | M.C.; Ponsioen, Cyriel; Oldenburg, Bas; Weersma, Rinse

---

VERSION 1 - REVIEW

**Reviewer**	**1**
**Name**	**Lewandowski, Konrad**
**Affiliation**	**National Medical Institute of the Ministry of the Interior and Administration, Gastroenterology**
**Date**	**23-Aug-2025**
**COI**	**None**

The article presents the study protocol for TURN2, a multicenter randomized, placebo-controlled trial evaluating the efficacy of fecal microbiota transplantation in patients with mild-to-moderate active ulcerative colitis. This study follows the earlier TURN trial, which suggested that clinical response to FMT was associated with specific bacterial strains and butyrate production. TURN2 aims to improve FMT efficacy through donor selection based on microbiota profile, strict anoxic stool processing to preserve obligate anaerobes, and repeated dual-route administration combining nasoduodenal and rectal delivery after bowel cleansing. The trial includes 76 patients with the possibility of expanding to 100. Patients receive four weekly FMTs, two by dual-route and two rectal only. The control group receives autologous FMT prepared in the same manner to maintain blinding. The primary endpoint is steroid-free clinical and endoscopic remission at week 8 assessed by the adapted Mayo score. Secondary endpoints include clinical response, endoscopic and histological remission, sustained remission at week 52, safety, and extensive microbiome and metabolite analyses. The study is monitored by an independent Data Safety Monitoring Board and has been registered in Dutch and international registries.

The manuscript has several important strengths. It addresses a clinically relevant problem, since current treatments for ulcerative colitis have limited long-term efficacy and carry significant risks. The scientific rationale is well grounded in prior work including the authors' own TURN trial, as well as evidence highlighting the role of butyrate-producing bacteria and microbial diversity. The protocol is innovative, combining donor selection, anaerobic stool preparation, and dual-route repeated administration, which differentiates it from earlier studies. The methodology is thoroughly described, with clear criteria for patient and donor selection, stool preparation, randomization, blinding, safety monitoring, and data handling. Endpoints are comprehensive, ranging from clinical and endoscopic outcomes to microbiome, metabolite, immune, and dietary analyses, which will yield valuable mechanistic insights. Ethical and safety standards are high, with extensive donor screening, quarantine and retesting protocols, and careful adverse event monitoring.

There are also limitations. Using autologous FMT as placebo, although justified for blinding, may reduce the ability to detect differences because previous studies have shown relatively high response rates in this comparator arm. Recruitment has been difficult due to COVID-19 and stricter donor regulations, which highlights practical challenges for clinical implementation. The intervention itself is complex, requiring dual-route administration, bowel preparation, and strict anaerobic processing, which may limit feasibility in routine practice. The high level of laboratory and logistical resources required, along with stringent donor criteria, reduces generalizability to non-academic settings and raises concerns about scalability and cost.

To strengthen the manuscript, it would be useful to expand the discussion on the implications of using autologous FMT as placebo, including how this may affect interpretation of efficacy. A table comparing previous randomized controlled trials of FMT in UC, outlining design, donor strategy, delivery method, and outcomes, would help place TURN2 in context. It would also be valuable to clarify which aspects of the protocol are most likely to be translatable to clinical practice and which remain experimental. Expanding the section on biomarkers of response could highlight the potential for personalized approaches in future.

In conclusion, this is a well-prepared and methodologically robust protocol with strong scientific justification and potential to provide meaningful contributions to the field. Despite practical limitations regarding feasibility and generalizability, the study design is sound and the level of detail is commendable. I recommend acceptance after minor editorial revisions, particularly to strengthen the discussion on limitations, the interpretation of the placebo arm, and clinical applicability.

**Reviewer**	**2**
**Name**	**Bao, Chunhui**
**Affiliation**	**Shanghai University of Traditional Chinese Medicine**

Date 05-Sep-2025

COI None

This is a well-written and timely study protocol for the TURN2 trial, which aims to improve the efficacy of fecal microbiota transplantation (FMT) in ulcerative colitis (UC) through strict anoxic stool processing, donor selection, and dual-route administration. The protocol is comprehensive, addresses important gaps in previous studies, and has the potential to contribute valuable insights into the optimization of microbial therapies. However, there are several methodological and reporting issues that require clarification or improvement before the protocol can be considered for publication.

1. The primary endpoint is steroid-free clinical and endoscopic remission at week 8. While this is rigorous, the rationale for choosing 8 weeks as the main time point is not sufficiently discussed. Please explain how short-term remission correlates with long-term outcomes and why 8 weeks is clinically meaningful.

2. Using autologous FMT improves blinding but may not be a true placebo, as it could have biological effects. The high placebo response rate in your earlier TURN trial supports this concern. Please provide more justification for this choice and discuss its impact on the ability to detect a treatment effect.

3. The assumed treatment effect (37%) and control remission rate (10%) appear optimistic. Please provide stronger justification with references or sensitivity analyses showing what happens if the control group rate is higher (e.g., 15–20%).

4. The statistical test described for the primary outcome is a two-sided Student's t-test. As the outcome is binary (remission yes/no), logistic regression or chi-square tests would be more appropriate. This needs correction.

5. A very large number of secondary and exploratory endpoints are listed, spanning clinical, endoscopic, histological, microbiome, metabolomic, immune, and dietary domains. This breadth is valuable but risks data dilution and false positives. Please clarify which secondary outcomes are core versus exploratory, and provide more detail on the planned adjustment for multiple testing (beyond stating "FDR correction").

6. The donor selection strategy based on microbial composition is innovative, but complex. Please provide data on anticipated donor acceptance rates and feasibility, as this may significantly affect trial progress.

7. The statistical analysis plan briefly mentions modified ITT analysis but provides limited details on how missing data will be handled (e.g., multiple imputation vs complete case). Please expand this section.

8. The dual-route administration (nasoduodenal + enema) and repeated bowel preparations are burdensome. Please elaborate on strategies to ensure adherence and how tolerability will be monitored.

9. Some sentences in the background and discussion are very long, making them difficult to follow. Consider shortening or restructuring for clarity.

VERSION 1 - AUTHOR RESPONSE

Reviewer: 1

Dr. Konrad Lewandowski, National Medical Institute of the Ministry of the Interior and Administration

Comments to the Author:
The article presents the study protocol for TURN2, a multicenter randomized, placebo-controlled trial evaluating the efficacy of fecal microbiota transplantation in patients with mild-to-moderate active ulcerative colitis. This study follows the earlier TURN trial, which suggested that clinical response to FMT was associated with specific bacterial strains and butyrate production. TURN2 aims to improve FMT efficacy through donor selection based on microbiota profile, strict anoxic stool processing to preserve obligate anaerobes, and repeated dual-route administration combining nasoduodenal and rectal delivery after bowel cleansing. The trial includes 76 patients with the possibility of expanding to 100. Patients receive four weekly FMTs, two by dual-route and two rectal only. The control group receives autologous FMT prepared in the same manner to maintain blinding. The primary endpoint is steroid-free clinical and endoscopic remission at week 8 assessed by the adapted Mayo score. Secondary endpoints include clinical response, endoscopic and histological remission, sustained remission at week 52, safety, and extensive microbiome and metabolite analyses. The study is monitored by an independent Data Safety Monitoring Board and has been registered in Dutch and international registries.

The manuscript has several important strengths. It addresses a clinically relevant problem, since current treatments for ulcerative colitis have limited long-term efficacy and carry significant risks. The scientific rationale is well grounded in prior work including the authors' own TURN trial, as well as evidence highlighting the role of butyrate-producing bacteria and microbial diversity. The protocol is innovative, combining donor selection, anaerobic stool preparation, and dual-route repeated administration, which differentiates it from earlier studies. The methodology is thoroughly described, with clear criteria for patient and donor selection, stool preparation, randomization, blinding, safety monitoring, and data handling. Endpoints are comprehensive, ranging from clinical and endoscopic outcomes to microbiome, metabolite, immune, and dietary analyses, which will yield valuable mechanistic insights. Ethical and safety standards are high, with extensive donor screening, quarantine and retesting protocols, and careful adverse event monitoring.

There are also limitations. Using autologous FMT as placebo, although justified for blinding, may reduce the ability to detect differences because previous studies have shown relatively high response rates in this comparator arm. Recruitment has been difficult due to COVID-19 and stricter donor regulations, which highlights practical challenges for clinical implementation. The intervention itself is complex, requiring dual-route administration, bowel preparation, and strict anaerobic processing, which may limit feasibility in routine practice. The high level of laboratory and logistical resources required, along with stringent donor criteria, reduces generalizability to non-academic settings and raises concerns about scalability and cost.

1. To strengthen the manuscript, it would be useful to expand the discussion on the implications of using autologous FMT as placebo, including how this may affect interpretation of efficacy.

We agree that the use of autologous FMT as comparator warrants explicit discussion. In the Discussion paragraph that starts with 'Creating a reliable placebo for FMT is challenging…' we already explain the rationale and implications for choosing autologous FMT as comparator in interpreting efficacy. Specifically, we state that autologous FMT was chosen to optimize blinding. We also acknowledge that autologous FMT is unlikely to be completely biologically inert, as seen in the TURN trial, and therefore may lead to a conservative estimate of the 'true' effect size of donor FMT, which now has been added to this paragraph. However, to overcome at least part of this effect, we deliberately used a stricter composite primary endpoint in TURN2 to reduce placebo/autologous response compared with the TURN trial. Our sample size and interim monitoring strategy are based on detecting a clinically meaningful difference between two potentially active interventions rather than between an active treatment and an inert placebo.

2. A table comparing previous randomized controlled trials of FMT in UC, outlining design, donor strategy, delivery method, and outcomes, would help place TURN2 in context.

We thank the reviewer for this valuable comment. Our study group conducted an extensive systematic review comparing previous trials on FMT in UC, including randomized controlled trials. We published this article in 2024 in Clinical Gastroenterology and Hepatology;

Bénard MV, de Goffau MC, Blonk J, Hugenholtz F, van Buuren J, Paramsothy S, Kaakoush NO, D'Haens GRAM, Borody TJ, Kamm MA, Ponsioen CY. Gut Microbiota Features in Relation to Fecal Microbiota Transplantation Outcome in Ulcerative Colitis: A Systematic Review and Meta-Analysis. Clin Gastroenterol Hepatol. 2025 Sep;23(10):1719-1736. doi: 10.1016/j.cgh.2024.10.001. Epub 2024 Oct 21. PMID: 39442743.

Within this review we also evaluated and reported the differences in study design of these studies, including RCTs, in Supplementary Table 1. Regarding (double-blinded and non-blinded) randomized controlled trials, the applied definitions of the primary outcomes and actual outcomes are outlined in Supplementary Table 4.

We added this reference in the discussion section on page 15 in the second paragraph. In addition, we added another systematic review evaluating the study designs and outcomes of 168 FMT studies in the same line.

This approach, we believe, gives readers a clear contextualization of the TURN2 study while avoiding redundant material and staying within the scope of a protocol paper.

3. It would also be valuable to clarify which aspects of the protocol are most likely to be translatable to clinical practice and which remain experimental.

We agree with the reviewer that it is useful for readers and clinicians to understand which components of the TURN2 protocol could reasonably be implemented in clinical practice and which should currently be regarded as experimental.

In the discussion we have added clarification about the administration and processing of the current protocol which we believe are translatable, although we acknowledge that this requires a specialized infrastructure (vacuum boxes, oxygen scavengers, rapid transfer to an anaerobic chamber), which is added in the subsection starting with: ' We designed a highly…' However, in its current format donor selection can indeed pose a problem for further generalizability as outlined in the discussion under paragraph starting with 'As recently elaborated…'. Therefore, in our view, the trial is designed both to test an 'optimized' FMT protocol and to generate evidence that may allow future simplification into more practical, standardised microbial therapeutics for clinical use. This last point has been added within this paragraph.

4. Expanding the section on biomarkers of response could highlight the potential for personalized approaches in future.

We agree with the reviewer that the protocol via its secondary outcomes entails a rich biomarker programme with the potential for personalised FMT or microbiota-based therapie. We have emphasized this by adding a sentence regarding the implication of these biomarkers for personalized approaches at the end of the subsection starting with: 'As recently elaborated…'.

In conclusion, this is a well-prepared and methodologically robust protocol with strong scientific justification and potential to provide meaningful contributions to the field. Despite practical limitations regarding feasibility and generalizability, the study design is sound and the level of detail is commendable. I recommend acceptance after minor editorial revisions, particularly to strengthen the discussion on limitations, the interpretation of the placebo arm, and clinical

applicability.

Reviewer: 2
Dr. Chunhui Bao
Comments to the Author:
This is a well-written and timely study protocol for the TURN2 trial, which aims to improve the efficacy of fecal microbiota transplantation (FMT) in ulcerative colitis (UC) through strict anoxic stool processing, donor selection, and dual-route administration. The protocol is comprehensive, addresses important gaps in previous studies, and has the potential to contribute valuable insights into the optimization of microbial therapies. However, there are several methodological and reporting issues that require clarification or improvement before the protocol can be considered for publication.

1. The primary endpoint is steroid-free clinical and endoscopic remission at week 8. While this is rigorous, the rationale for choosing 8 weeks as the main time point is not sufficiently discussed. Please explain how short-term remission correlates with long-term outcomes and why 8 weeks is clinically meaningful.

The reason for choosing the primary endpoint at 8 weeks stems from our earlier extensive experience with metabolic syndrome and FMT in UC (Vrieze et al. Gastroenterology 2012; Rossen et al. Gastroenterology 2017). After one nasoduodenal administration of donor feces the altered microbiota signature tends to recert to its original composition after 4 weeks. With repeated nasoduodenal administration as was done in the TURN trial several patients responded early within one week, but in some favorable and durable response was only observed after 6 weeks. This is why in TURN2 we chose to delineate the primary endpoint at 8 weeks. Most responders at the primary endpoint in the TURN trial were still in clinical remission at 52 weeks. We added this information to the methods section.

2. Using autologous FMT improves blinding but may not be a true placebo, as it could have biological effects. The high placebo response rate in your earlier TURN trial supports this concern. Please provide more justification for this choice and discuss its impact on the ability to detect a treatment effect.

We thank the reviewer for this comment. This has been addressed as well by reviewer 1 and addressed accordingly (see above) and added within the discussion under the subsection ‘Creating a reliable placebo for FMT is challenging…’.

3. The assumed treatment effect (37%) and control remission rate (10%) appear optimistic. Please provide stronger justification with references or sensitivity analyses showing what happens if the control group rate is higher (e.g., 15–20%).

We agree with the reviewer that this is an optimistic treatment effect, however justified based on the four published RCTs published at that time. See this acknowledged in the sentence: “The other three published RCTs had efficacy rates from 24 to 32% and placebo rates from 5 to 9%.” Since we are using an optimized protocol also compared to these trials we assume a slightly higher efficacy rate. The justification via references has been added within this paragraph.

4. The statistical test described for the primary outcome is a two-sided Student’s t-test. As the outcome is binary (remission yes/no), logistic regression or chi-square tests would be more appropriate. This needs correction.

We apologize for this error and corrected our statistical test, see page 13.

5. A very large number of secondary and exploratory endpoints are listed, spanning clinical, endoscopic, histological, microbiome, metabolomic, immune, and dietary domains. This breadth is valuable but risks data dilution and false positives. Please clarify which secondary outcomes are core versus exploratory, and provide more detail on the planned adjustment for multiple testing (beyond stating “FDR correction”).

We appreciate this important point and have revised this in our manuscript. All listed secondary outcomes are in our view core outcomes, however, we agree that microbiome, metabolomic, immune and dietary analysis are explorative. We added this difference in the subtitles. With regard to multiple testing, we have revised the subsection of statistical analysis and added specifically the Holm-Bonferroni procedure for correction of multiple testing of the secondary outcomes.

We further emphasize that the trial is powered on the primary endpoint; core secondary outcomes provide clinically relevant supporting evidence; and exploratory analyses are mainly intended to identify patterns and candidate biomarkers for future studies.

6. The donor selection strategy based on microbial composition is innovative, but complex. Please provide data on anticipated donor acceptance rates and feasibility, as this may significantly affect trial progress.

Our study group, together with other FMT researchers within the Amsterdam UMC published the following article on donor acceptance rates;

Bénard MV, de Bruijn CMA, Fenneman AC, Wortelboer K, Zeevenhoven J, Rethans B, Herrema HJ, van Gool T, Nieuwdorp M, Benninga MA, Ponsioen CY. Challenges and costs of donor screening for fecal microbiota transplantations. PLoS One. 2022 Oct 20;17(10):e0276323. doi: 10.1371/journal.pone.0276323. PMID: 36264933; PMCID: PMC9584411.

We refer to this article in the section ‘’donors’’.

7. The statistical analysis plan briefly mentions modified ITT analysis but provides limited details on how missing data will be handled (e.g., multiple imputation vs complete case). Please expand this section.

We thank the reviewer for the comment. We now expanded how to handle missing data in this section.

8. The dual-route administration (nasoduodenal + enema) and repeated bowel preparations are burdensome. Please elaborate on strategies to ensure adherence and how tolerability will be monitored.

At week 0 and week 3 (the dual-route administration days), study staff record the volume of bowel preparation ingested (in litres of Moviprep). Incomplete intake of Moviprep, or incomplete administration of standard FMT volumes, is documented in the electronic case report form as a protocol deviation. During nasoduodenal placement and enema administration, the trained nurse and physicians provide reassurance and support, and patients are encouraged—but not forced—to retain enemas as long as they comfortably can. We record and document the retention time of the FMT enemas. Patients are asked about discomfort related to the nasoduodenal tube and rectal administration, and any symptoms were recorded as adverse events when applicable. All adverse events and serious adverse events, including those related to bowel preparation or the administration routes, are monitored throughout the study and reviewed by the independent DSMB.

We have elaborated on this point by adding a separate paragraph in the FMT section of the Methods, providing details on guidance and support by trained staff, recording of bowel preparation and FMT administration, encouragement of enema retention, and monitoring of discomfort and adverse events at each visit.

9. Some sentences in the background and discussion are very long, making them difficult to follow. Consider shortening or restructuring for clarity.

Thank you for this comment, we have checked the manuscript and where appropriate have rewritten the sentences
